# Vertebral osteomyelitis secondary to *Streptococcus cristatus* infection

**DOI:** 10.1016/j.heliyon.2023.e19616

**Published:** 2023-09-02

**Authors:** Helen Tran, Angela Ai, Oscar E. Gallardo-Huizar, Michael Kahn, Glenn Mathisen

**Affiliations:** a Department of Medicine, Olive View-UCLA Medical Center, Sylmar, CA, United States; b Division of Infectious Diseases, Olive View-UCLA Medical Center, Sylmar, CA, United States

**Keywords:** *Streptococcus cristatus*, *Vertebral osteomyelitis*

## Abstract

A 66-year-old male with a history of low back pain was found to have discitis and osteomyelitis. Biopsy and PCR testing revealed *Streptococcus cristatus* infection. This bacteria does not typically cause disease, and only a few cases in the literature have reported it to cause infection in the bones or joints. This case illustrates that vertebral osteomyelitis with a rare causative agent, *S. cristatus*, is possible and can be identified with PCR. Treatment typically requires long-term antibiotics tailored to the causative agent for a minimum of 6 weeks and can sometimes include surgical management.

## 1 Introduction

*Streptococcus cristatus* belongs to the *Streptococcus mitis* group and is a commensal Gram-Positive, catalase-negative cocci that grows in chains and is typically found in the oral microbiota of humans [[1], [2], [3], [4]]*.* Previous studies have shown that they are related to *S. sanguis* via DNA-DNA hybridization [5,6]. However, Jensen et al. [7] concluded that *S. cristatus* and *oligofermentans* were synonymous via clustering patterns seen on genomic analysis. This bacteria has been reported to cause endocarditis and septicemia [4,8]. Still, there are a few reported cases of it causing bone and joint infections, except for one report of septic arthritis in a neonate [3]. Here we present a case of vertebral osteomyelitis due to S. cristatus in a 66-year-old man with low back pain.

## 2 Case

A 65-year-old male with a history of myasthenia gravis (MG), hypertension (HTN), chronic kidney disease (CKD), and diabetes (DM) presented to a community hospital with 40 pounds of weight loss over three months, cough without hemoptysis, and low back pain. He denied fevers or chills. His physical exam was notable for mild left flank tenderness. Notable labs on admission included blood urea nitrogen (BUN) that was >200 mg/dL with creatinine of >11 mg/dL (previously less than 1 mg/dL in 2017), elevated lipase with normal liver function tests, hemoglobin 7.8 g/dL, and a positive COVID polymerase chain reaction (PCR). Initial imaging was notable for a chest x-ray showing pulmonary consolidations at the medial and posterior-bilateral infrahilar zones. Subsequent computerized tomography (CT) abdomen and pelvis without contrast showed severe bilateral hydroureteronephrosis with an enlarged prostate, nonspecific bilateral perinephric stranding, and possible destructive changes at L4 and L5 with a differential of discitis, osteomyelitis, or severe degenerative joint disease (DJD). Of note, he also had a maxillofacial CT showing periodontal disease. Patient was supposed to be on mycophenolate and pyridostigmine for his myasthenia gravis, but had stopped taking his medications.

An MRI without contrast of the lumbar spine had findings consistent with discitis and osteomyelitis at the L4–L5 level without evidence of epidural phlegmon or abscess, edema in bilateral posterior paravertebral muscles from the L4 level through the sacrum, likely reactive and representing myositis, and myositis involving bilateral psoas muscles at the L4–L5 level without abscess (Image A). Subsequent infectious workup revealed a false-positive cocci IgG, positive quantiferon gold without evidence of negative active tuberculosis, and a positive urine culture for group B streptococcus. Additionally, the patient had an elevated erythrocyte sedimentation rate (ESR) at 119 mm/h and C-reactive protein (CRP) at 93 mg/L. There was a high suspicion of osteomyelitis, so concerted efforts were made to have the patient undergo a CT-guided spinal biopsy with bacterial, fungal, and AFB cultures sent, in addition to being sent for the University of Washington universal PCR test (UW uPCR). Pathology revealed acute and chronic inflammation with micro abscess formation, necrosis, and rare hemosiderin-laden histiocytes. Cultures did not grow organisms, but the UW uPCR eventually resulted with *S. cristatus.* He was subsequently started on oral amoxicillin for an 8-week course. It was thought that our patient likely seeded his vertebrae through his mouth, given that *S. cristatus* is an oral commensal bacterium and the patient had periodontal disease found on maxillofacial CT.

The patient was followed up outpatient at six months and reported an overall improvement in his symptom and completed his course of antibiotics without any complications or new symptoms. Repeat ESR was 60 mm/hr, and CRP was 11 mg/L.

**Image A:**
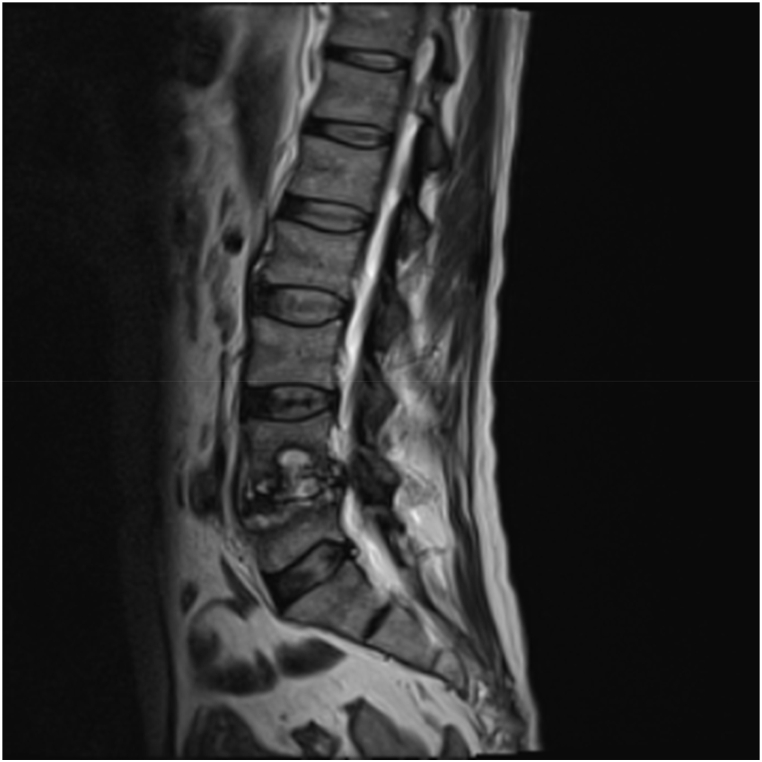
Lumbar Spine Magnetic Resonance Imaging (MRI) showing discitis osteomyelitis at the L4–L5 level without evidence of epidural phlegmon or abscess. No spinal canal stenosis. Also, severe bilateral neural foraminal stenosis at L4–L5.

## 3 Discussion

*S. cristatus* is a Gram-Positive cocci that often grows with other oral streptococci species in periodontal biofilms. It is typically found as part of the commensal human oral mucosal microbiota and was initially isolated in 1991 [1]. This bacteria rarely cause disease, though a few reports of bacteremia and endocarditis have been reported in the literature [[9], [10], [11], [12]]. More commonly, it has been suggested in the literature as a dental pathogen and found in dental caries [13,14]. To our knowledge, there have not been any reports of *S. cristatus* causing any bone infection except for a case of septic arthritis in a neonate reported by Gupta et al. [3].

Vertebral osteomyelitis usually arises from either the spread of infection through the bloodstream or directly extending from the skin. The primary causative organism is Staph aureus, but other organisms like enteric Gram-negative bacilli, pyogenic and non-pyogenic streptococci, and Pseudomonas can also be involved. The oral microbiota can also cause transient bacteremia and endocarditis. It has been well-reported that dental extractions and poor dental hygiene are related to bacteremia and endocarditis [8,15]. This is due to routine activities such as tooth brushing, flossing, chewing, and endodontic procedures causing mucosal and gingival trauma, allowing bacteria to enter the bloodstream. Bacteremia can be transient and asymptomatic, causing no sequelae in healthy individuals who are not immunocompromised. However, in patients with diseased valves, the bacteremia can seed the valves, causing persistent bacteremia and further infection throughout the body, including the spine. The microbiota in the mouth has been identified as Gram-negative bacteria, including *P. gingivalis, Capnocytophaga, and Fusobacterium,* as well as Gram-positive bacteria such as Viridans group streptococci [15]. Interestingly, he did not have endocarditis seen on transesophageal echocardiogram (TEE), despite most cases of Strep cristatus infection being reported in the setting of endocarditis [[9], [10], [11], [12]].

Vertebral osteomyelitis is typically diagnosed with spinal imaging, typically MRI if available [16]. If imaging findings are consistent with osteomyelitis, the patient should have a CT-guided needle biopsy of the bone and disc space that is sent for culture and Gram staining [16]. Our patient's bone culture did not grow any organisms; however, UW uPCR confirmed *Strep cristatus,* which was the apparent cause of his osteomyelitis Management of vertebral osteomyelitis entails long-term antibiotics tailored to the culture of the biopsy for a minimum of 6 weeks and outpatient monitoring for soft tissue extension or cord compression. While awaiting cultures, patients can be given empiric antibiotics to cover common causes of vertebral osteomyelitis, including staphylococci, streptococci, and Gram-Negative bacilli. This case demonstrates the value of PCR for diagnosis of vertebral osteomyelitis—in addition to identifying a likely pathogen (with subsequent successful treatment), the PCR was negative for mycobacteria and fungi, definite concerns in light of the positive Quantiferon Gold and +IgG for coccidioidomycosis. In our case, our patient received eight weeks of amoxicillin with clinical improvement, confirming the likely etiology of *Strep cristatus*.

## 4 Conclusion

This case highlights that vertebral osteomyelitis with *S. cristatus*, an organism regularly thought of as commensal human oral mucosa microbiota, is possible. It also emphasizes the importance of culture and PCR in determining the causative organism and guiding antibiotic therapy.

## Patient consent

Informed consent was obtained from the patient(s) (or relative/guardian) for the publication of all images, clinical data and other data included in the manuscript.

## Author contribution statement

All authors listed have significantly contributed to the investigation, development and writing of this article.

## Data availability statement

No data was used for the research described in the article.

## Declaration of competing interest

The authors declare that they have no known competing financial interests or personal relationships that could have appeared to influence the work reported in this paper.
